# Network pharmacology and experimental verification of the potential mechanism of Er-Xian decoction in aplastic anemia

**DOI:** 10.1038/s41598-023-44672-9

**Published:** 2023-10-13

**Authors:** Mei Ye, Guangxian Liu, Yujun Yang, Hongyu Yang, Juan Ren, Wenfei Chen, Zeli Gao

**Affiliations:** 1 Department of Hematology, The Affiliated Hospital of Panzhihua University, Panzhihua, China; 2 Department of Pharmacy, The Affiliated Hospital of Panzhihua University, Panzhihua, China; 3 School of Basic Medicine, Panzhihua University, Panzhihua, China; 4 Department of Clinical Laboratory, The Affiliated Hospital of Panzhihua University, Panzhihua, China

**Keywords:** Therapeutics, Pharmacology

## Abstract

To investigate the potential mechanism of Er-Xian decoction (EXD) in treating aplastic anemia (AA), the active components of EXD were screened by the Traditional Chinese Medicine Systems Pharmacology Database and Analysis Platform (TCMSP), and the targets of the components were predicted by the Swiss Target Prediction database. AA targets were collected from the GeneCards, OMIM, DisGeNET, PharmGKB, DrugBank, and TTD databases, the intersection of AA targets and EXD targets was calculated, and an herb-component-target network was constructed by Cytoscape 3.7.2 software. The STRING database was used for protein‒protein interaction (PPI) analysis, and Cytoscape 3.7.2 software was used to construct a PPI network and perform topology analysis. The core targets were imported into the DAVID database for gene ontology (GO) and Kyoto Encyclopedia of Genes and Genomes (KEGG) pathway analyses. The molecular docking software AutoDock was used to measure the affinity between active components and key targets. Finally, we established a mouse model of AA and verified the key targets and signaling pathways of EXD by RT‒PCR, ELISA and Western blot analysis. A total of 53 active components were screened from EXD, 2516 AA-related targets were collected, and 195 common targets were obtained. An herb-component-target network and a PPI network were successfully constructed, and 36 core targets were selected from the PPI network. The main active components of EXD include luteolin, kaempferol, berberine, etc., and key targets include PIK3CA, AKT1, STAT3, etc. GO functional enrichment analysis showed that cell components, molecular functions and biological processes with significant correlations were macromolecular complexes, protein serine/threonine/tyrosine kinase activity and protein phosphorylation, respectively. KEGG pathway analysis showed that the pathways with significant correlations included the PI3K-Akt signaling pathway and JAK-STAT signaling pathway. Molecular docking results showed that the tested key targets had good affinity for the corresponding active components. In AA mice, we found that EXD significantly increased white blood cell count, red blood cell count, platelet count and hemoglobin levels, increased mRNA levels of PIK3CA, PIK3CD, AKT1, JAK2, STAT3 and MAPK1, and promoted phosphorylation of PI3K, AKT, ERK1/2 and STAT3. In summary, EXD acts on PI3K, AKT, STAT3 and other targets through berberine, luteolin, quercetin and other components to regulate the PI3K-Akt pathway, JAK-STAT pathway and other pathways, thus exerting its therapeutic effect on AA. This study explained the Chinese medicine theory of treating AA with EXD by tonifying kidney-yang and provides a scientific basis for the use of EXD in treating AA.

## Introduction

Aplastic anemia (AA) is a rare immune-mediated hematopoietic disorder characterized by weakened proliferation of bone marrow hematopoietic cells, reduced peripheral whole blood cells, and immune dysfunction^1^. The main clinical manifestations consist of different degrees of anemia, hemorrhage and infection with significant morbidity and mortality^2^. At present, the pathogenesis of AA has not been fully elucidated, and it is generally believed that AA is related to hyperimmune function, a defective hematopoietic microenvironment, a shortage or dysfunction of hematopoietic stem cells, and genetic abnormalities^3^. The incidence of AA is significantly higher in East Asia than in other regions, especially among young people^4^. AA can be divided into very severe aplastic anemia (VSAA), severe aplastic anemia (SAA), and nonsevere aplastic anemia (NSAA) according to severity in the Camitta standard and into acute AA and chronic AA according to the progression of the disease in China^5,6^. For young patients with immune AA, bone marrow transplantation is the preferred treatment, which can achieve satisfactory results^7,8^. Immunosuppressive therapy is appropriate for all patients, especially those who are not candidates for bone marrow transplantation, but late consequences of the disease may occur because it cannot replace the affected bone marrow or immune system^1^. Other treatment strategies include androgen and stem cell stimulation, but the results are often less than satisfactory. Therefore, searching for an alternative and more effective treatment of AA is necessary.

In traditional Chinese medicine (TCM) theory, there is no corresponding disease name of AA, which can be classified into the category of “deficiency and strain” (Xulao in Chinese), “bone strain” (Gulao in Chinese), “blood deficiency” (Xuexu in Chinese), and “blood depletion” (Xueku in Chinese) according to its clinical manifestations, and it is thought that the lesion is mainly in the bone marrow, involving the spleen, kidney and liver^9,10^. There are two views on the pathogenesis of AA in Chinese medicine: deficiency-caused AA (Yin-Xu-Zhi-Bing in Chinese) and poison-caused AA (Yin-Du-Zhi-Bing in Chinese), which correspond to immune AA and acquired AA in general^9^. The theory of deficiency-caused AA posits that deficiency and damage to kidney essence leads to depression of kidney Yang (Shen-Yang-Xu in Chinese), which cannot stimulate bone marrow hematopoiesis, resulting in blood deficiency^11^. Chinese medicine posits that the kidney dominates the bone and generates the marrow (Zhugu-Shengsui in Chinese), so tonifying the kidney and warming the kidney Yang is the fundamental strategy to treat AA^10,11^.

Er-Xian decoction (EXD) is a classic TCM formulation comprising 6 herbs: Rhizoma Curculiginis (Xianmao in Chinese, XM), Epimedii Folium (Yinyanghuo in Chinese, YYH), Radix Angelicae Sinensis (Danggui in Chinese, DG), Radix Morindae Officinalis (Bajitian in Chinese, BJT), Cortex Phellodendri Chinensis (Huangbo in Chinese, HB), and Rhizoma Anemarrhenae (Zhimu in Chinese, ZM)^12^. EXD possesses the effect of warming kidney Yang (Wen-Shen-Yang in Chinese) and tonifying kidney essence (Bu-Shen-Jing in Chinese) and is widely used in the clinical treatment of menopausal syndrome^13^. Modern scientific studies have shown that EXD also has many therapeutic effects, such as anti-osteoporosis, ameliorating myocardial damage during menopause, repairing spinal cord injury and anti-depressant effects^14–17^. Based on the kidney-tonifying effect of EXD and the traditional Chinese medicine theory of tonifying the kidney in the treatment of AA, we believe that EXD can be used to treat AA. In fact, clinical studies have shown that EXD has a significant curative effect on AA. A clinical study of 84 AA patients treated with EXD showed that the total effective rate of EXD could reach 84.5%. Another study showed that the use of EXD in addition to cyclosporine and stanozolol significantly improved bone marrow hematopoietic function and reduced complications^18,19^. Some herbs or ingredients in EXD have also been used alone to improve AA pathology; for example, DG polysaccharide prevents mitochondrial apoptosis by regulating the Treg/Th17 ratio in aplastic anemia, and icariin in YYH can improve the hematopoietic function of cyclophosphamide-induced myelosuppression mice^20,21^. In the current study, we observed the therapeutic effect of EXD on AA mice and explored the mechanism using network pharmacology combined with experimental validation.

## Materials and methods

### Network pharmacology analysis

#### Active ingredient screening and target prediction

The Traditional Chinese Medicine Systems Pharmacology Database and Analysis Platform (TCMSP, https://old.tcmsp-e.com/tcmsp.php) was used to search the chemical ingredients of XM, YYH, DG, BJT, HB, and ZM in EXD^22^. The active ingredients were screened according to oral bioavailability (OB) > 30% and drug-likeness (DL) > 0.18, and the ingredients without PubChem CID were removed. All active ingredients were queried canonical SMILES by the PubChem database (https://pubchem.ncbi.nlm.nih.gov), and then the putative targets were predicted by the Swiss Target Prediction database according to the canonical SMILES.

#### AA-related target collection

AA-related targets were searched in GeneCards, OMIM, DisGeNET, PharmGKB, DrugBank, and Therapeutic Target Database (TTD) using “aplastic anemia” as a key word^23–28^. The combined set of targets from six databases served as the AA target library.

#### Component-target network construction

The intersection of AA targets and EXD targets was identified by the Venny2.1 platform (https://bioinfogp.cnb.csic.es/tools/venny), which represents potential targets for EXD treatment of AA. The component-target network was constructed using active components and potential targets by Cytoscape 3.7.2 software^29^.

#### Protein‒protein interaction (PPI) analysis

The potential targets of EXD for AA were imported into the STRING database (https://cn.string-db.org), the organism was set as “*Homo sapiens*”, the minimum required interaction score was set as 0.7, other parameters were set as default, and PPI analysis was performed^30^. Topology analysis was used to measure the importance of nodes, including three parameters: degree, betweenness, and closeness.

#### Gene ontology (GO) and Kyoto encyclopedia of genes and genomes (KEGG) pathway enrichment analyses

To reveal the multiple mechanisms of EXD treatment of AA, potential targets were used for GO functional and KEGG pathway enrichment analyses using the Database for Annotation, Visualization and Integrated Discovery (DAVID, https://david.ncifcrf.gov)^31^. In brief, the gene symbols of potential targets were imported into DAVID, the organism was set as “*Homo sapiens*”, and GO functional and KEGG pathway enrichment analyses were performed^32,33^.

#### Molecular docking

Virtual molecular docking was employed to evaluate the affinity between the active compounds and the key targets of EXD in the treatment of AA. The structure of the target was obtained from the RCSB Protein Data Bank (RCSB PDB, https://www.rcsb.org/), and the structure of the active compound was downloaded from the PubChem database (https://pubchem.ncbi.nlm.nih.gov/). After pretreatment by PyMOL software or Chem3D software, the active compound and the target were imported into AutoDock software for docking. The Lamarckian genetic algorithm (LGA) was used to find the best binding state between the ligand (active compound) and receptor (target), and energy matching was used to evaluate the binding ability between the ligand and receptor.

### Experimental verification

#### Preparation of EXD

All 6 kinds of Chinese herbs in EXD were purchased from Sichuan Chinese herbal medicine Co., LTD (Chengdu, Sichuan Province, China) and identified as genuine by Professor Guangxian Liu of the Affiliated Hospital of Panzhihua University. The EXD was prepared by improving the previous method^16^. In brief, the herbs XM, YYH, DG, BJT, HB and ZM were mixed at a ratio of 12:12:10:10:9:9, and the mixture (500 g) was ground into a powdered form. The constituents were extracted twice with 5 L of double-distilled water at 100 °C for 0.5 h each time. Then, the mixed extract was filtered and concentrated at 50 °C, lyophilized to yield EXD powder, and then stored at − 20 °C for subsequent use. By calculation, the extraction rate of EXD from herbs is approximately 20.31%.

#### Animals and treatment

Male and female BALB/c mice (6 weeks old and weighing 18–22 g) were purchased from Chengdu Dossy Experimental Animal Co., LTD [license number: SCXK (Chuan) 2015–030] (Chengdu, Sichuan, China). Mice were housed under specific pathogen-free conditions and cared for in accordance with the guidelines of the National Science and Technology Committee of China. All procedures and animal experiments were approved by the Animal Care and Use Committee of Panzhihua University.

A total of 50 mice were randomly divided equally into five groups (each consisting of five females and five males): normal, AA, low-dose EXD (EXDL), medium-dose EXD (EXDM), and high-dose EXD (EXDH). Except for the normal group, all mice in the other groups were used to establish the AA model according to a previous method^34^. Briefly, the mice were exposed to 6.0 Gy total body irradiation at approximately 1.0 Gy/min and injected via the tail vein with two million lymphocytes isolated from the lymph glands of DBA mice within 4 h after irradiation. Seventy-two hours after irradiation, intragastric administration was started, the normal group and AA group were given normal saline, and the EXDL, EXDM, and EXDH groups were given the corresponding doses of EXD (100, 200, and 400 mg/kg d^−1^, respectively, equivalent to doses of native Chinese herbs of 0.5, 1, and 2 g/kg d^−1^)^16^. All animals were treated continuously for 21 days. All mice were killed by cervical dislocation after treatment.

#### Peripheral blood routine examination

After treatments, peripheral blood was collected from the fundus venous plexus of mice for routine analysis, including white blood cell count (WBC), red blood cell count (RBC), platelet count (PLT) and hemoglobin (HB).

#### Real-time PCR

After the mice were sacrificed, the femur was removed, and normal saline was used with a syringe to flush out the bone marrow cells for PCR analysis. Total RNA was isolated from bone marrow cells using an RNAsimple Total RNA Kit (TIANGEN, Beijing, China) according to the manufacturer’s instructions. The RNA was reverse-transcribed into cDNA using a HiScript II 1st Strand cDNA Synthesis Kit (Vazyme, Nanjing, Jiangsu, China), and then AceQ Universal SYBR qPCR Master Mix (Vazyme, Nanjing, Jiangsu, China) was used to perform real-time PCR using cDNA as a template. Using GAPDH as a reference gene, the relative expression level of the target gene was calculated according to the comparative Ct method. The primers used in the experiment were as follows: forward sequence CACCTGAACAGACAAGTAGAGGC and reverse sequence GCAAAGCATCCATGAAGTCTGGC for PI3KCA, forward sequence ACCATCAGTGGCTCTGCGGTTT and reverse sequence GTGGTCTTCTGGGAACTCACCT for PI3KCD, forward sequence GGACTACTTGCACTCCGAGAAG and reverse sequence CATAGTGGCACCGTCCTTGATC for AKT1, forward sequence GCTACCAGATGGAAACTGTGCG and reverse sequence GCCTCTGTAATGTTGGTGAGATC for JAK2, forward sequence TCAAGCCTTCCAACCTCCTGCT and reverse sequence AGCTCTGTACCAACGTGTGGCT for MAPK1, forward sequence AGGAGTCTAACAACGGCAGCCT and reverse sequence GTGGTACACCTCAGTCTCGAAG for STAT3, and forward sequence CATCACTGCCACCCAGAAGACTG and reverse sequence ATGCCAGTGAGCTTCCCGTTCAG for GAPDH.

#### Western blotting

Western blot assays were carried out according to previously described methods^35^. Briefly, bone marrow cells were lysed on ice with RIPA solution for 20 min and then centrifuged at 12,000 r/min at 4 °C for 10 min. The supernatant was mixed with 5 × loading buffer, heated at 95 °C for 5 min, shock cooled and mixed for SDS gel electrophoresis. Then, the protein in the gel was transferred to a PVDF membrane and blocked with 5% skim milk TBST solution at room temperature for 1 h. It should be noted that before membrane transfer, the PVDF membrane of appropriate size only covered the gel of the target protein region, so the blot image cannot show the complete gel. The 5% skim milk TBST solution was discarded, and the membrane was washed 3 times with TBST. Then, the primary antibodies, including p-PI3K, PI3K, p-AKT, AKT, p-STAT3, STAT3, p-ERK1/2, ERK1/2, and β-actin (Cell Signaling Technology, Danvers, MA, United States), were added and incubated at 4 °C overnight. The next day, the primary antibody was discarded and washed 3 times with TBST, and the secondary antibody was added and incubated at room temperature for 1 h. An ECL chemiluminescent reagent (Vazyme, Nanjing, Jiangsu, China) was used to develop protein bands on the membrane. These bands were quantitatively analyzed by ImageJ software (National Institutes of Health, Bethesda, MD, United States).

### Statistical analysis

The statistical analysis in this study included two aspects: the P values of the GO and KEGG enrichment analyses in the network pharmacological analysis were completed by the DAVID database, and the data obtained from the experiment were expressed as the mean ± standard deviation (SD). GraphPad Prism 8.0 software (La Jolla, CA, USA) was used for statistical analysis and mapping. For experimental data, significant differences between groups were assessed using one-way ANOVA followed by Tukey’s multiple comparison test or two-way ANOVA followed by Sidak’s multiple comparisons test. *P* < 0.05 was considered statistically significant.

### Ethical approval

We state that all experiments in this study follow the guidelines for ARRIVE and comply with ethical requirements. The animal experiments in this study followed the Guidelines for Ethical Animal Welfare (2018 edition) of the National Science and Technology Committee of China and were approved by the Experimental Animal Welfare Ethics Committee of the Affiliated Hospital of Panzhihua University.

## Results

### EXD active ingredient collection, target prediction and herb-component-target network construction

**Figure 1:**
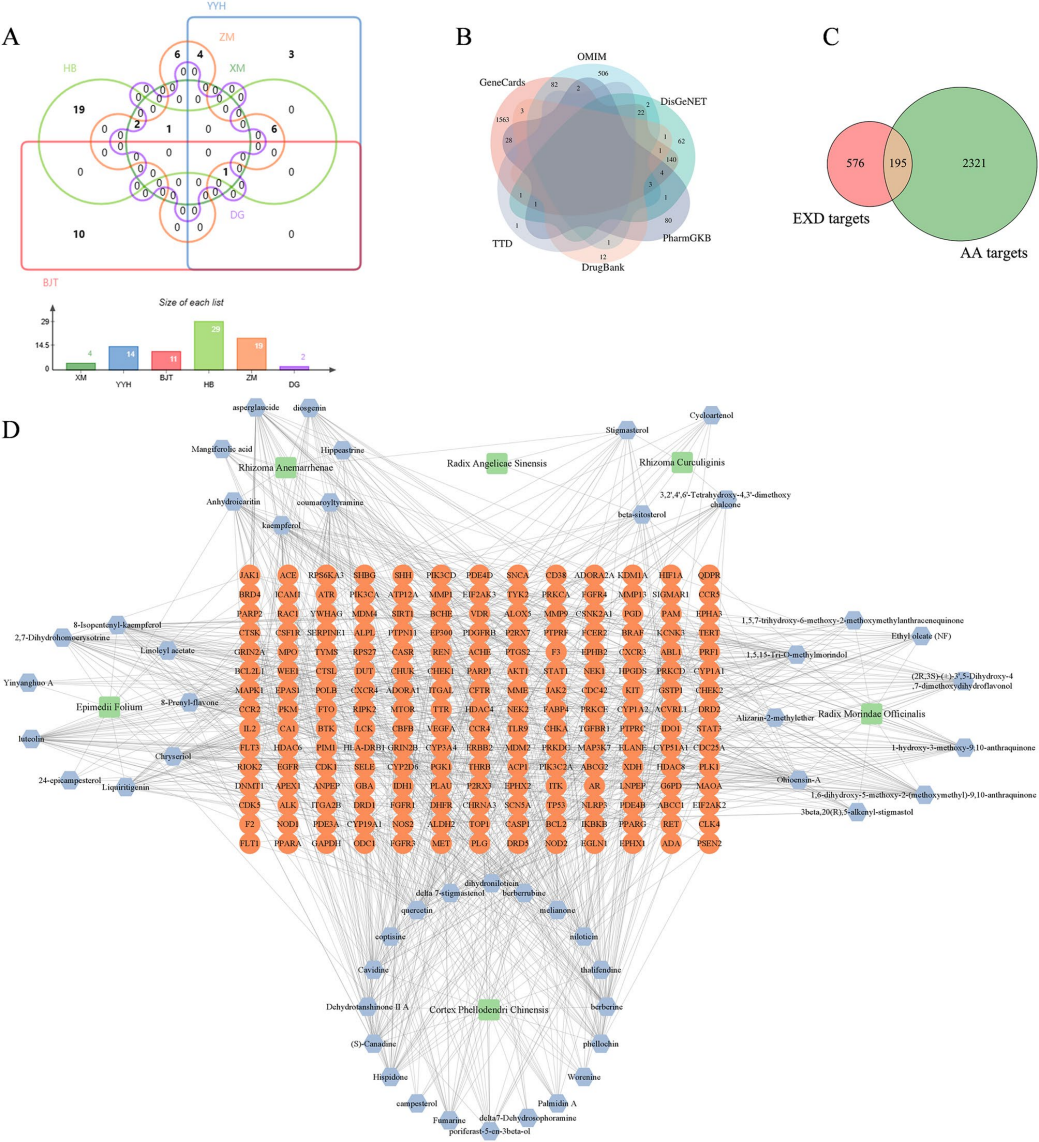
VENN diagrams of EXD ingredients and AA-related targets and herb-component-target network. (**A**) Venn diagram of the herbal ingredients of EXD. (**B**) Venn diagram of AA-related targets collected from the GeneCards, OMIM, DisGeNET, PharmGKB, DrugBank and TTD databases. (**C**) Venn diagram of AA targets and EXD targets. (**D**) Herb-component-target network map of EXD in the treatment of AA.

According to the established screening criteria, 4 ingredients were collected from XM, 14 from YYH, 2 from DG, 12 from BJT, 23 from HB, and 9 from ZM (Table S1). Beta-sitosterol is the common component of XM, YYH, DG, BJT and HB, stigmasterol is the common component of XM, DG, HB and ZM, quercetin and poriferast-5-en-3beta-ol are the common components of YYH and HB, and kaempferol and anhydroicaritin are the common components of YYH and ZM (Fig. 1A). After removing the duplicate ingredients, 53 active ingredients were obtained, and their 771 predicted targets were identified. A total of 2516 AA-related targets were collected, some of which were shared by multiple databases (Fig. 1B). The Venn diagram showed that AA and EXD shared 195 common targets, suggesting that these targets may be potential targets of EXD in the treatment of AA (Fig. 1C). We used Cytoscape software to construct an herb-component-target network to describe the interactions between herbs and their constituents and potential targets (Fig. 1D), and topological analysis showed that the 10 active ingredients with the highest degree values were luteolin, kaempferol, chryseriol, coumaroyltyramine, quercetin, hispidone, berberine, 1,5,15-tri-O-methylmorindol, 1,6-dihydroxy-5-methoxy-2-(methoxymethyl)-9,10-anthraquinone, and cavidine.

### PPI network analysis

**Figure 2:**
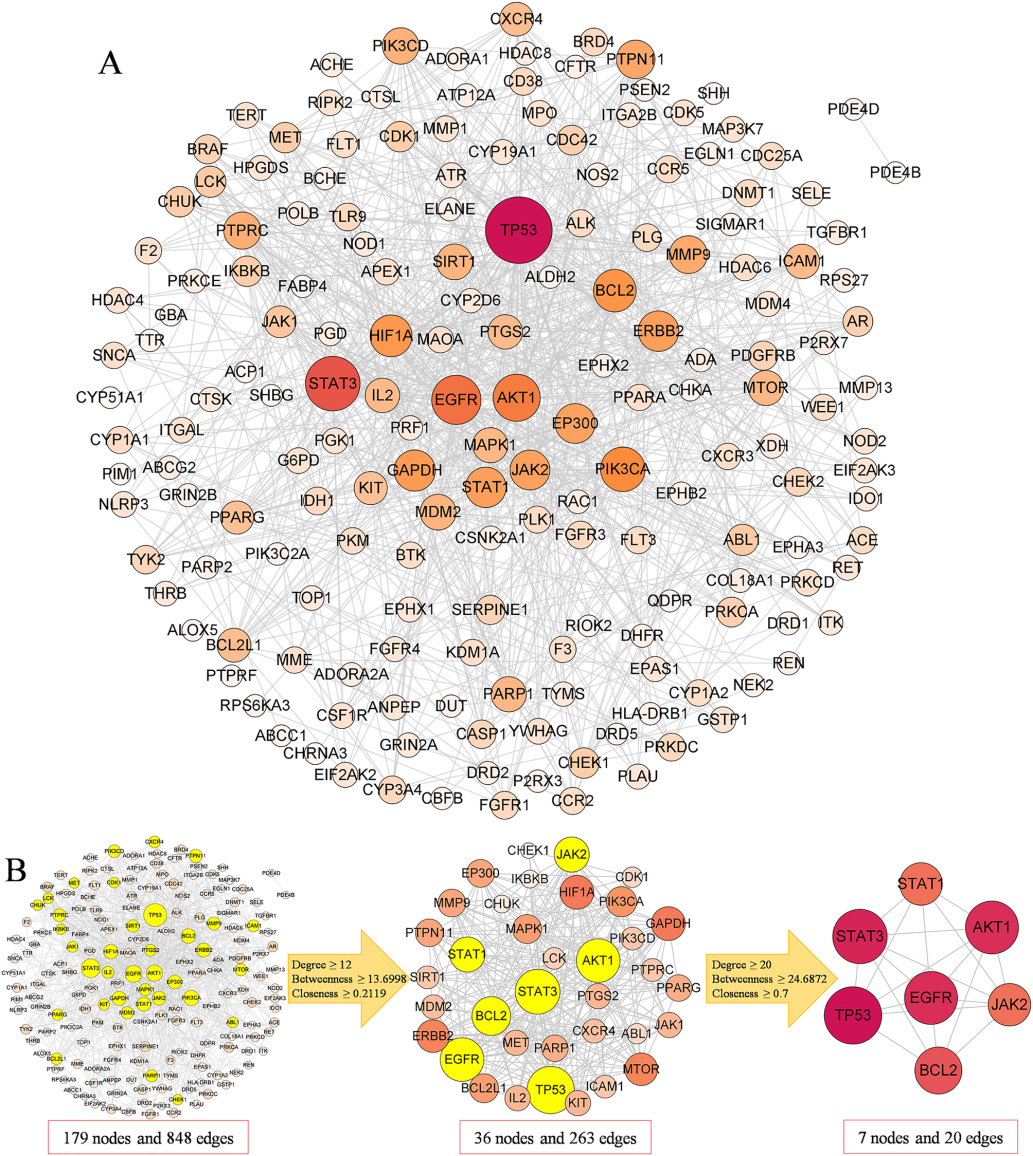
PPI network and core target screening process. (**A**) PPI network map of potential targets for EXD treatment of AA. Since there are 16 targets with no interaction under high confidence conditions, only 179 targets are shown in the PPI network map. (**B**) Core target and key target screening process.

To explore the interactions between 195 potential targets, we imported these targets into the STRING database for PPI analysis and imported PPI data into Cytoscape software to build a PPI network. The PPI network has 179 nodes and 848 edges, representing 179 potential targets and 848 pairs of interaction relations, respectively. The larger the area of the node and the darker the color, the larger the degree value of the node and the more important it is (Fig. 2A). Under the high confidence condition, there were 16 potential targets with no interaction relationship. We filtered the PPI network twice based on topological data, which included degree, betweenness and closeness (Table S2). In the first filtering, a core target network with 36 nodes and 263 edges was obtained based on the double median degree value. In the second filtering, a key target network with 7 targets and 20 edges was obtained. Therefore, the PPI analysis results suggest that TP53, STAT3, AKT1, EGFR, BCL2, JAK2 and STAT1 may be the key targets of EXD in treating AA (Fig. 2B).

### GO functional and KEGG pathway enrichment analysis

**Figure 3:**
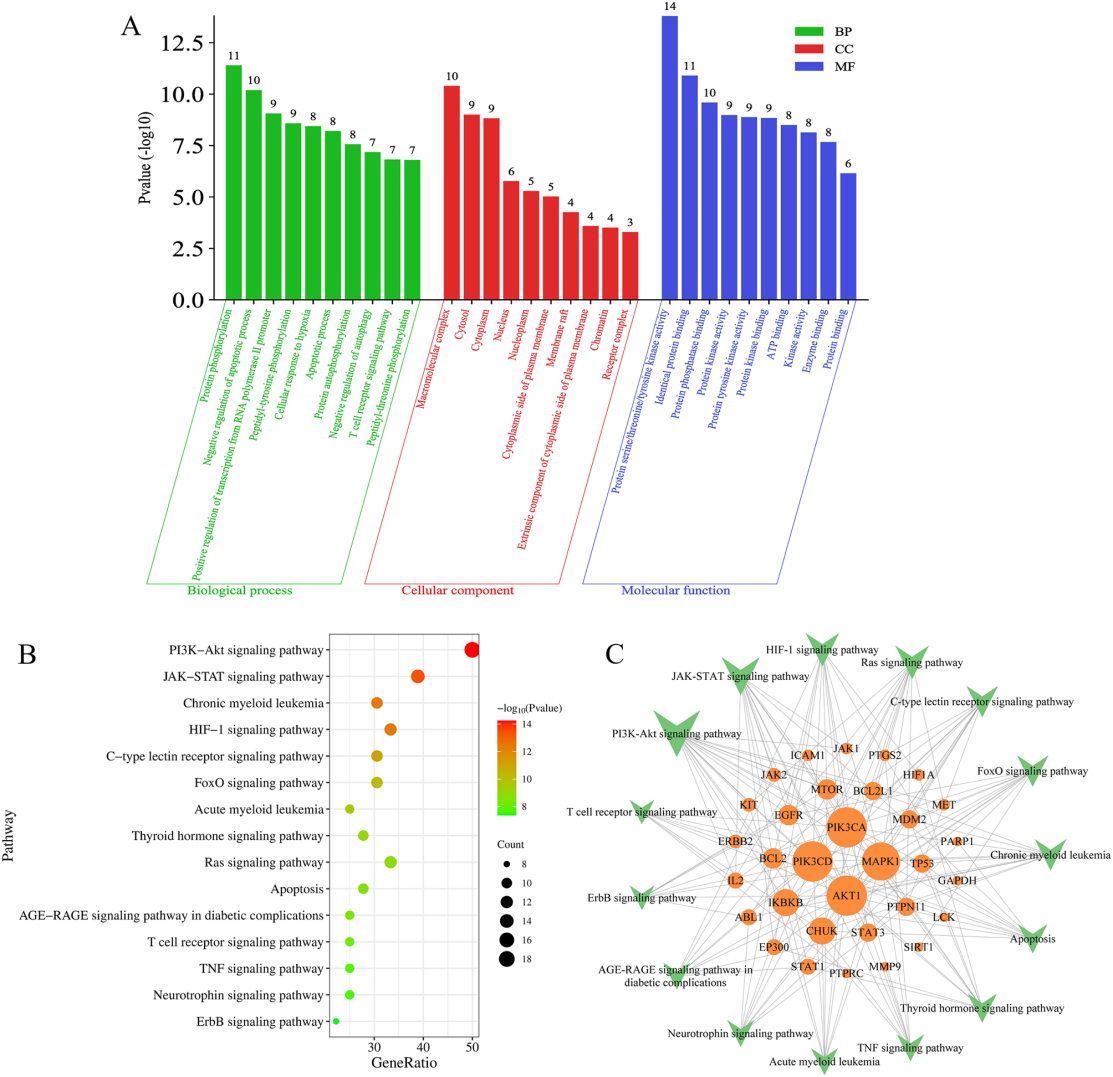
GO functional and KEGG pathway enrichment analyses of core targets. (**A**) Bar diagram of GO functional enrichment analysis. (**B**) Bubble diagram of the top 15 KEGG pathways with the lowest P values. (**C**) The network diagram of the top 15 KEGG pathways with the lowest P value and corresponding core targets.

The 36 core targets were used for GO functional and KEGG pathway enrichment analysis. In brief, the gene symbols of 36 core targets were imported into the DAVID database, and the species was set as *Homo sapiens* for GO functional and KEGG pathway enrichment analyses, including cellular component (CC), molecular function (MF), and biological process (BP). A total of 343 GO terms with P < 0.05 were enriched, including 33 CC terms, 58 MF terms, and 252 BP terms. We selected the top 10 terms for significance, as shown in Fig. 3A and Table S3. The results showed that the molecular regulatory mechanism of EXD treatment of AA was mainly carried out in the macromolecular complex, cytosol, cytoplasm and so on, and the main molecular functions involved included protein serine/threonine/tyrosine kinase activity, identical protein binding, protein phosphatase binding and so on. The biological processes involved were mainly protein phosphorylation, negative regulation of the apoptotic process, positive regulation of transcription from the RNA polymerase II promoter and so on. The results of KEGG pathway enrichment analysis showed that 36 core targets were enriched in 122 pathways with *P* < 0.05, and Fig. 3B and Table S4 shows the 15 pathways with the highest significance, including the PI3K-Akt signaling pathway, JAK-STAT signaling pathway, chronic myeloid leukemia, etc. To further study the correlation between these pathways and core targets, we constructed a target-pathway network, and topological analysis showed that PI3KCA, PI3KCD, AKT1 and MAPK1 had the highest degree value, suggesting that these targets play an important role in the treatment of AA by EXD (Fig. 3C).

### Molecular docking

**Figure 4:**
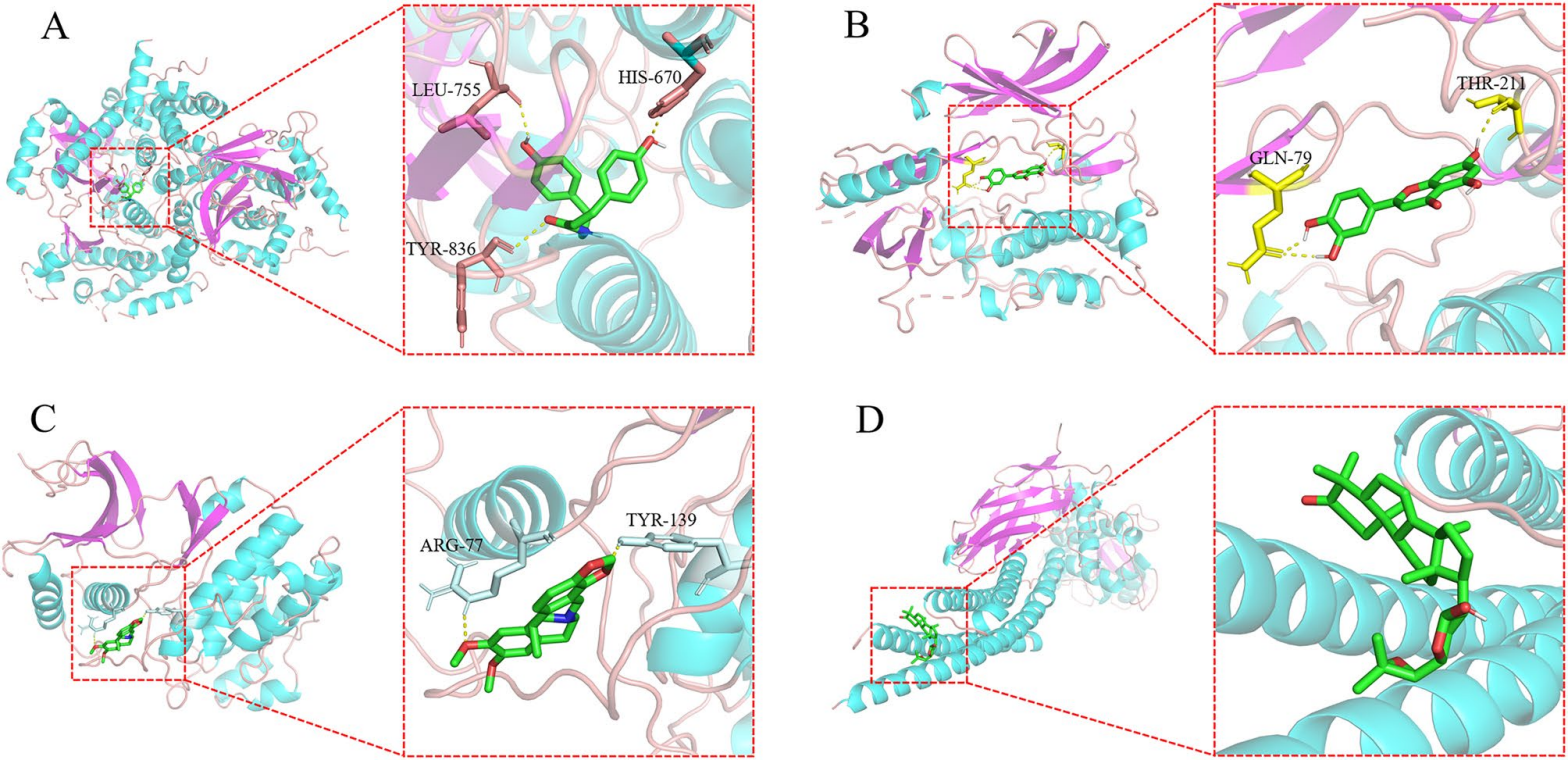
Three-dimensional diagram of binding sites between active compounds and key targets. (**A**) Diagram of binding sites between coumaroyltyramine and PI3Kα. (**B**) Diagram of binding sites between luteolin and AKT1. (**C**) Diagram of binding sites between cavidine and ERK2. (**D**) Diagram of binding sites between melianone and STAT3.

Molecular docking was used to simulate the binding between the active ingredients of EXD and the key targets. Combined with the results of PPI network analysis and target-pathway network analysis, we selected PI3Kα (PIK3CA), AKT1, ERK2 (MAPK1) and STAT3 as the tested macromolecules to examine their binding energy with their corresponding active compounds. The results showed that coumaroyltyramine can form hydrogen bonds with LEU-755, HIS-670 and TYR-836 residues of PI3Kα, and the binding energy was − 4.82 kcal/mol; luteolin can form hydrogen bonds with GLN-79 and THR-211 residues of AKT1, and the binding energy was − 5.76 kcal/mol; and cavidine can form hydrogen bonds with ARG-77 and TYR-139 residues of ERK2, and the binding energy was − 6.07 kcal/mol (Fig. 4A–C). The binding energy of melianone with STAT3 was − 6.74 kcal/mol, but no hydrogen bond was found, and the binding energy may result from intermolecular forces (Fig. 4D). These results indicated that the tested compounds had good affinity for the target.

### EXD increased peripheral blood WBC, RBC, PLT and HB in AA mice

**Figure 5:**
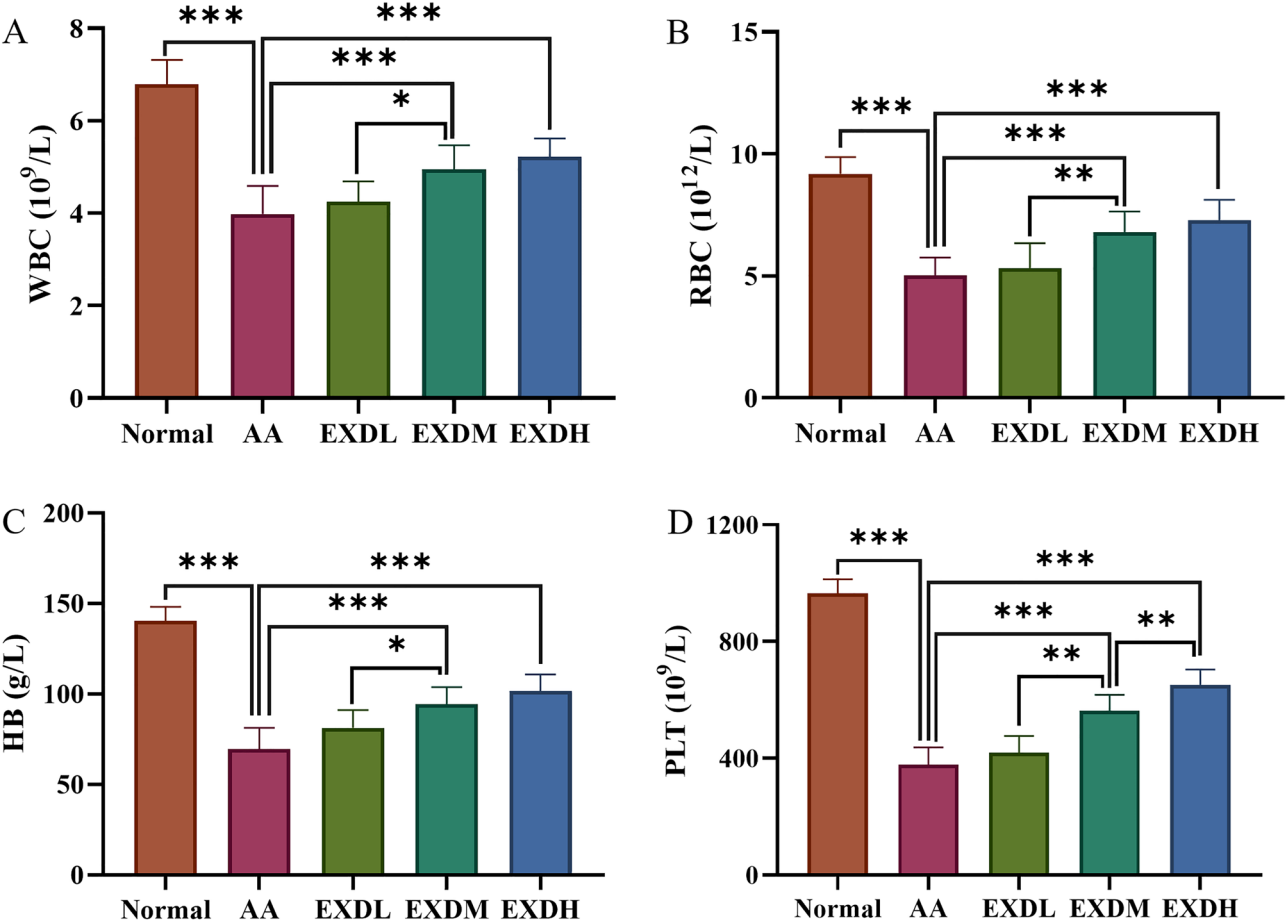
Effect of EXD on peripheral blood cell counts in AA mice. White blood cell count (**A**), red blood cell count (**B**), platelet count (**C**) and hemoglobin content (**D**) in the peripheral blood of AA mice. The data are expressed as the mean ± SD, ^*^P < 0.05, ^**^P < 0.01, ^***^P < 0.001.

To observe the effect of EXD on peripheral blood cell counts in AA mice, we first examined routine blood tests. The results showed that the WBC, RBC, PLT and HB of AA mice were significantly decreased compared with those of normal mice but significantly increased after the treatment of AA mice with EXD, suggesting that EXD is beneficial to the hematopoietic function of AA mice (Fig. 5).

### EXD increased PIK3CA, PIK3CD, AKT1, JAK2, MAPK1 and STAT3 mRNA levels in bone marrow cells of AA mice

**Figure 6:**
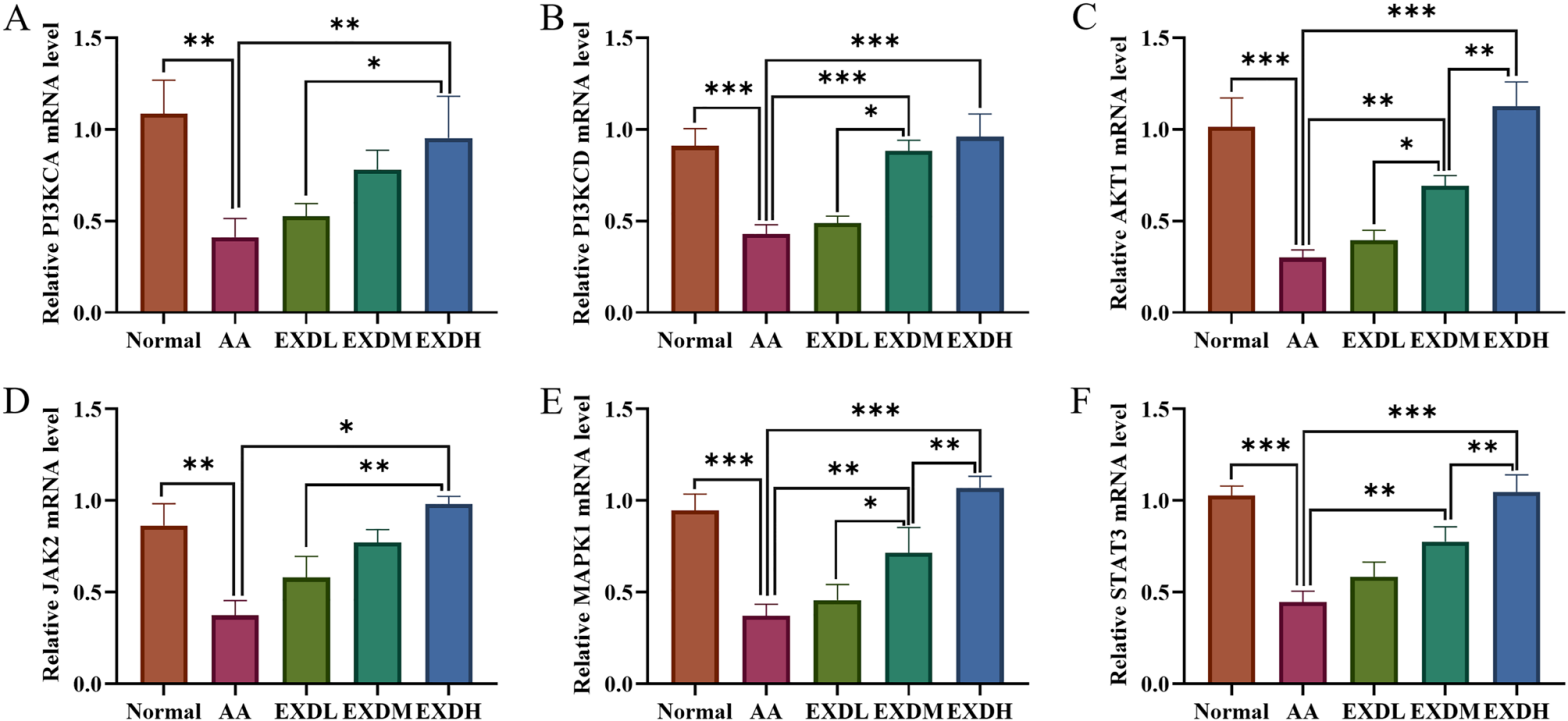
Effects of EXD on mRNA levels of PI3KCA, PIK3CD, AKT1, JAK2, MAPK1 and STAT3 in AA mouse bone marrow cells. (**A**) The mRNA level of PIK3CA. (**B**) The mRNA level of PIK3CD. (**C**) The mRNA level of AKT1. (**D**) The mRNA level of JAK2. (**E**) The mRNA level of MAPK1. (**F**) The mRNA level of STAT3. The data are expressed as the mean ± SD, ^*^P < 0.05, ^**^P < 0.01, ^***^P < 0.001.

Network pharmacological analysis showed that PI3KCA, PIK3CD, AKT1, JAK2, MAPK1 and STAT3 were the key targets of EXD in the treatment of AA. Therefore, the mRNA levels of PI3KCA, PIK3CD, AKT1, JAK2, MAPK1 and STAT3 in bone marrow cells of AA mice were detected by RT‒PCR. The results showed that the mRNA levels of the above genes were significantly decreased in AA mice but significantly increased in AA mice treated with EXD (Fig. 6). These results suggested that the therapeutic effect of EXD on AA might be related to the regulation of the expression of PI3KCA, PIK3CD, AKT1, JAK2, MAPK1 and STAT3.

### EXD activated the PI3K-AKT pathway and promoted the phosphorylation of STAT3 and ERK1/2

**Figure 7:**
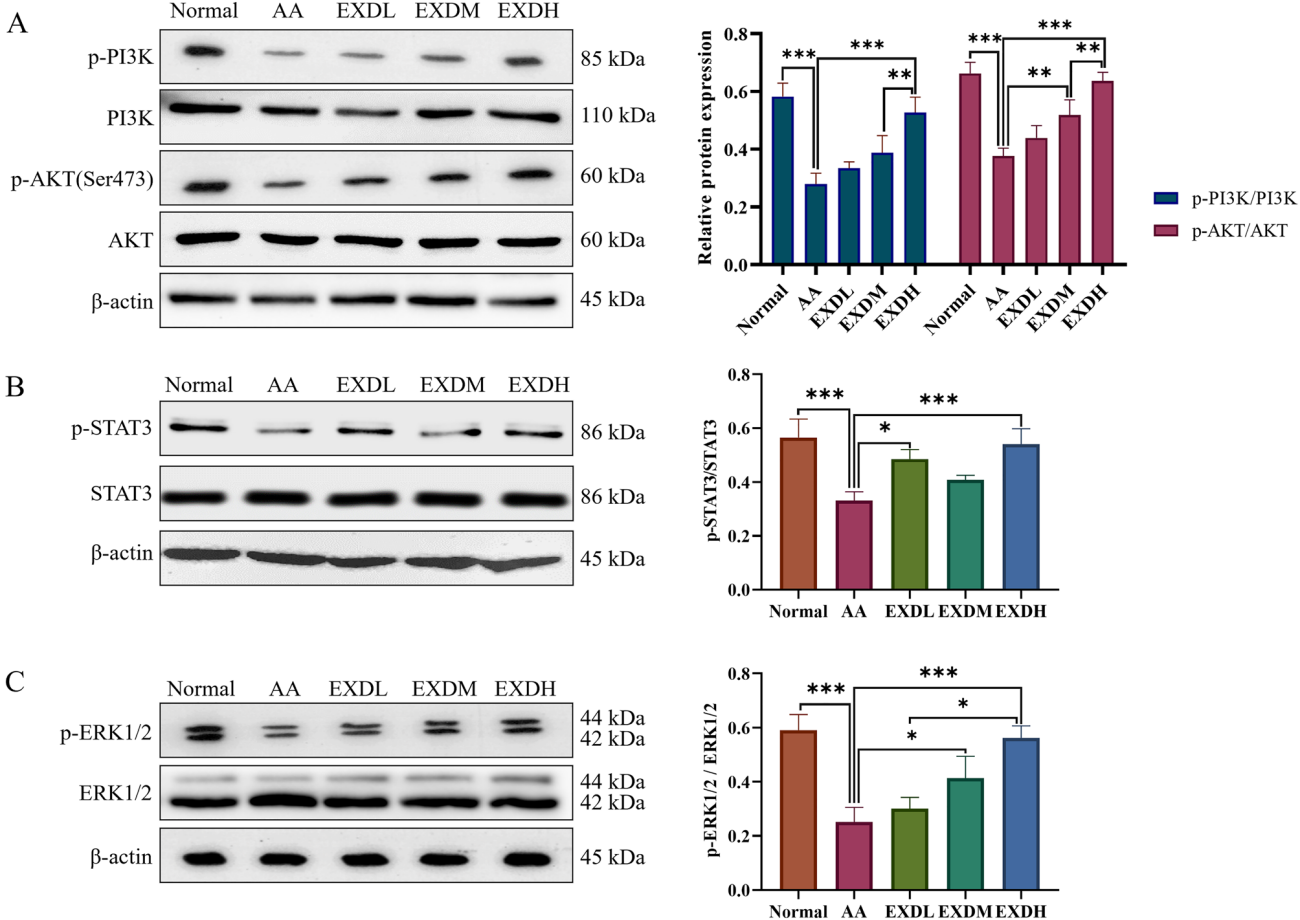
Effects of EXD on PI3K-AKT pathway activation and STAT3 and ERK1/2 phosphorylation. (**A**) Effect of EXD on the activation of the PI3K-AKT pathway. (**B**) Effect of EXD on the protein phosphorylation of STAT3. (**C**) Effect of EXD on the protein phosphorylation of ERK1/2. The data are expressed as the mean ± SD, ^*^P < 0.05, ^**^P < 0.01, ^***^P < 0.001.

To further verify the expression of key targets and the activation of signaling pathways of EXD in the treatment of AA, we detected the expression and phosphorylation of PI3K, AKT, STAT3 and ERK1/2. The results showed that the phosphorylation of PI3K and AKT in AA mice was lower than that in the normal group, and EXD could promote the phosphorylation of PI3K and AKT in a concentration-dependent manner, suggesting that EXD could activate the PI3K-Akt pathway (Fig. 7A). Compared with the normal group, the phosphorylation of STAT3 and ERK1/2 in the AA group was also significantly reduced, and EXD could promote the phosphorylation of STAT3 and ERK1/2, suggesting that EXD might activate downstream signals of STAT3 and ERK1/2 (Fig. 7B,C). The full-length images of western blot is shown in Figure S1.

## Discussion

Although there is no exact disease name corresponding to AA in Chinese medicine, AA is associated with deficiency in the traditional view^10^. The name “bone marrow deficiency” first appeared in *Huangdi's Canon of Medicine* and was described as “the Yin and Yang are exhausted, the blood and Qi are in deficiency”^9,10^. In the 1950s, the name and diagnostic criteria for AA were also applied in Chinese medicine, and AA was gradually included in the category of bone marrow deficiency in subsequent decades^36^. In recent decades, the most popular perspectives of Chinese medicine on the pathogenesis of AA include kidney essence deficiency, spleen-stomach weakness, and liver blood deficiency, among which the mainstream view is kidney essence deficiency^10^. Therefore, tonifying the kidney and warming Yang is the key to treating AA.

EXD is a prescription for tonifying the kidney and warming Yang and is widely used for diseases related to deficiency of kidney Yin and Yang^16,37^. In this study, we first investigated the main active components of EXD to clarify the material basis of EXD in the treatment of AA. Through the analysis of the herb-component-target network, we found that the top 10 compounds with the highest degree values were luteolin, kaempferol, chryseriol, coumaroyltyramine, quercetin, hispidone, berberine, 1,5,15-tri-O-methylmorindol, 1,6-dihydroxy-5-methoxy-2-(methoxymethyl)-9,10-anthraquinone, and cavidine. These 10 compounds acted on 109 of 195 potential targets, suggesting that these compounds may play an important role in the treatment of AA. Anti-inflammatory and antibacterial effects of 1,5,15-tri-O-methylmorindol and 1,6-dihydroxy-5-methoxy-2-(methoxymethyl)-9,10-anthraquinone have been reported, but more pharmacological activities have not been fully studied^38,39^. As a well-known natural product, berberine has a wide range of pharmacological activities, such as antibacterial, anti-inflammatory, antitumor, blood pressure regulation, metabolic regulation and neuroprotective effects^40,41^. Berberine can inhibit T helper cell activity by interfering with cytokine receptors or downstream signaling pathways^42^. This suggests that berberine weakens the immune system’s attack on hematopoietic cells through immunosuppression, which may contribute to the treatment of AA. Luteolin, quercetin and kaempferol are very common flavonoids that share similar chemical structures and have a wide range of biological activities, such as anti-inflammatory, antitumor and neuroprotective properties. Other studies have reported that oral gavage administration of luteolin alleviates anemia in HgCl_2_-treated rats, luteolin 7-glucoside guides hematopoietic stem cell differentiation into the erythroid lineage, and quercetin has a potential therapeutic effect on Fanconi anemia and inhibits protein and lipid oxidation on the erythrocyte membrane in Leishmania-infected animals, thereby arresting the development of anemia during the postinfection period^43–46^. Cavidine alleviates LPS-induced organ damage through antioxidant and anti-inflammatory activities^47,48^. The potential benefits of these compounds on hematopoietic function and inhibitory potential on immune function partly reflect the material basis of EXD treatment of AA.

We conducted PPI analysis of potential targets of EXD for AA treatment and found that AKT1, STAT3, EGFR, TP53, BCL2, JAK2 and STAT1 were the most important nodes in the PPI network after two screenings, suggesting that these seven genes play an important role in the treatment of AA by EXD. Previous studies have demonstrated that the PI3K/AKT signaling pathway, EGFR/ERK signaling pathway, and JAK-STAT signaling pathway promote hematopoietic cell proliferation^49–52^. GO and KEGG analyses showed that 252 biological processes and 122 signaling pathways were involved in the treatment of AA by EXD. GO functional analysis reflected that the most significant biological process was protein phosphorylation, the most significant cell component was macromolecular complex, cytosol, and cytoplasm, and the most significant molecular function was protein serine/threonine/tyrosine kinase activity. These results suggest that the mechanism of EXD treatment of AA is mainly related to the massive signal transmission occurring in the cytoplasm. KEGG pathway enrichment analysis showed that the PI3K/AKT signaling pathway was the most important pathway, and the JAK/STAT signaling pathway and MAPK signaling pathway were also enriched. Additionally, the core target-pathway network further reveals the importance of PI3K, AKT and MAPK. One of the most important signal transduction mechanisms in hematopoietic cells is the production of the lipid second messenger PIP3 regulated by PI3K^53^. PI3K converts PIP2 to PIP3, which leads to activation of the serine/threonine kinase AKT^54^. Activation of AKT promotes activation of downstream signals, which regulate the cell cycle, apoptosis, protein translation, autophagy, and metabolism^54,55^. Reduced PIP3 signaling impairs most aspects of hematopoiesis, including hematopoietic stem cell homeostasis and the development or function of T, B, and NK cells, myeloid mast cells, monocytes, granulocytes, and erythrocytes^53,56^. Therefore, activating the PI3K/AKT signaling pathway has positive significance for promoting hematopoietic development. The JAK/STAT pathway is another important signal regulating hematopoietic development. STAT3 is the only STAT family member that is essential for development; it plays a central role in the regulation of hematopoiesis and plays a critical role in the development of T helper cell and B-cell subsets as well as dendritic cell development and maturation^57^. Cytokines activate or inhibit STAT3 activity in hematopoietic progenitors to maintain moderate hematopoietic levels; however, hyperactivation or mutation of STAT3 can lead to tumor development^58,59^. ERK1 (also called MAPK3) and ERK2 (also called MAPK1) play crucial roles in cell survival, proliferation, migration, and differentiation in many tissues. Inhibition of ERK activation has been shown to hinder the transition of hematopoietic progenitor cells, and the absence of ERK1 and ERK2 in murine hematopoietic cells leads to bone marrow aplasia, leukopenia, anemia, and early lethality^60,61^.

To preliminarily verify the affinity between the active ingredients of EXD and the key targets, we simulated the binding situation by the molecular docking method, and the results showed that the tested compounds had good affinity with the target. To further verify the analysis results, we conducted experiments on an AA mouse model. The results showed that EXD significantly improved the peripheral blood cell count of AA mice, activated the PI3K/AKT pathway, and promoted the phosphorylation of STAT3 and ERK1/2. These data confirm the results of our network pharmacological analysis.

In conclusion, this study preliminarily revealed the main active ingredients and potential molecular mechanisms of EXD in the treatment of AA through network pharmacological analysis combined with animal experiments, providing a scientific basis for the use of EXD in the treatment of AA. However, this study also has some shortcomings. For example, this study collected the active ingredients of various herbs in EXD through a public database as the active ingredient cluster of EXD, which may be different from the real pharmacodynamic substances in EXD. In addition, the target prediction of active ingredients depends on the algorithm of the database, but the regulatory relationship between these components and corresponding targets has not been verified by experiments. Our study partially revealed the molecular mechanism of EXD treatment of AA, and a more comprehensive and in-depth mechanism needs further research.

## Conclusion

In the present study, we revealed the molecular mechanism by which EXD regulates the PI3K/AKT signaling pathway, JSK/STAT signaling pathway, and other pathways for AA treatment using network pharmacology and verified these mechanisms in an AA mouse model. This study uses modern scientific methods to clarify the effectiveness and mechanism of EXD in treating AA by warming Yang and tonifying the kidney (Wen-Yang-Bu-Shen in Chinese) and provides a scientific basis for the ethnopharmacological use of EXD.

### Supplementary Information

Supplementary Information.

## Data Availability

The original data for this study are available by contacting the corresponding authors.
