# Supplementary material for: Network pharmacology and experimental verification of the potential mechanism of Er-Xian decoction in aplastic anemia
Source: Sci Rep. 2023 Oct 13;13:17385. doi: 10.1038/s41598-023-44672-9 (PMC10575897; doi:10.1038/s41598-023-44672-9)
Supplement: Supplementary file 1 — Supplementary Information. [file 41598_2023_44672_MOESM1_ESM.pdf]

Supplementary Information

Network Pharmacology and Experimental Verification of the Potential Mechanism of Er-Xian Decoction in Aplastic Anemia

Mei Ye[1], Guangxian Liu[2], Yujun Yang[3], Hongyu Yang[4], Juan Ren[4], Wenfei Chen[2] and Zeli Gao[1, *]

[1] Department of Hematology, The Affiliated Hospital of Panzhihua University, Panzhihua, China
[2] Department of Pharmacy, The Affiliated Hospital of Panzhihua University, Panzhihua, China
[3] School of Basic Medicine, Panzhihua University, Panzhihua, China
[4] Department of Clinical Laboratory, The Affiliated Hospital of Panzhihua University, Panzhihua, China
[*] Corresponding author: Zeli Gao: gzllzg@126.com

Table S1. Active ingredients of EXD

NO.	Molecule Name	PubChem CID	Herb
EX01	beta-sitosterol	222284	XM, YYH, DG, BJT, HB
EX02	Stigmasterol	5280794	XM, DG, HB, ZM
EX03	Cycloartenol	92110	XM
EX04	3,2',4',6'-Tetrahydroxy-4,3'-dimethoxy chalcone	5321858	XM
EX05	luteolin	5280445	YYH
EX06	quercetin	5280343	YYH, HB
EX07	kaempferol	5280863	YYH, ZM
EX08	24-epicampesterol	5283637	YYH
EX09	Linoleyl acetate	5319042	YYH
EX10	poriferast-5-en-3beta-ol	457801	YYH, HB
EX11	Liquiritigenin	114829	YYH
EX12	Chryseriol	5280666	YYH
EX13	8-Isopentenyl-kaempferol	5318624	YYH
EX14	Anhydroicaritin	5318980	YYH, ZM
EX15	2,7-Dihydrohomoerysotrine	296195	YYH
EX16	Yinyanghuo A	154497711	YYH
EX17	8-Prenyl-flavone	17861868	YYH
EX18	Ethyl oleate (NF)	5363269	BJT
EX19	Alizarin-2-methylether	80103	BJT
EX20	1,5,15-Tri-O-methylmorindol	16203753	BJT
EX21	1,5,7-trihydroxy-6-methoxy-2-methoxymethylanthracenequinone	91477408	BJT
EX22	1,6-dihydroxy-5-methoxy-2-(methoxymethyl)-9,10-anthraquinone	11674044	BJT
EX23	1-hydroxy-3-methoxy-9,10-anthraquinone	13412786	BJT
EX24	1-hydroxy-6-hydroxymethylanthracenequinone	44595036	BJT

EX25	2-hydroxy-1,8-dimethoxy-7-methoxymethylanthracenequinone	86099557	BJT
EX26	(2R,3S)-(+)-3',5-Dihydroxy-4 ,7-dimethoxydihydroflavonol	5316762	BJT
EX27	3beta,20(R),5-alkenyl-stigmastol	7067828	BJT
EX28	Ohioensin-A	442531	BJT
EX29	Palmidin A	163048779	HB
EX30	Fumarine	4970	HB
EX31	phellamurin_qt	101643010	HB
EX32	berberine	2353	HB
EX33	(S)-Canadine	21171	HB
EX34	coptisine	72322	HB
EX35	delta 7-stigmastenol	12315385	HB
EX36	Dehydrotanshinone II A	128994	HB
EX37	delta7-Dehydrosophoramine	162869976	HB
EX38	dihydroniloticin	73351981	HB
EX39	niloticin	44559946	HB
EX40	Worenine	20055073	HB
EX41	Cavidine	193148	HB
EX42	Hispidone	21603613	HB
EX43	berberrubine	72703	HB
EX44	campesterol	12358798	HB
EX45	melianone	44575793	HB
EX46	phellochin	14021541	HB
EX47	thalifendine	3084288	HB
EX48	diosgenin	99474	ZM
EX49	coumaroyltyramine	13939145	ZM
EX50	asperglaucide	13855373	ZM
EX51	Mangiferolic acid	45270099	ZM
EX52	Chrysanthemaxanthin	162948692	ZM
EX53	Hippeastrine	441594	ZM

Table S2. Topological analysis of PPI network

NO.	Target	Degree	Betweenness	Closeness
1	TP53	68	7325.2314	0.260615
2	STAT3	50	2389.2053	0.250352
3	EGFR	42	2805.913	0.250704
4	AKT1	39	1649.7657	0.247566
5	PIK3CA	35	1094.5228	0.238926
6	BCL2	32	1235.89	0.243836
7	HIF1A	31	914.3918	0.239892
8	GAPDH	30	1953.6288	0.245517
9	STAT1	29	928.2633	0.242838
10	ERBB2	29	1446.5667	0.24152
11	JAK2	28	679.0654	0.235139

12	EP300	28	978.5512	0.233903
13	PTPN11	26	794.01825	0.229381
14	MMP9	25	1808.474	0.238286
15	PTPRC	24	738.61865	0.225316
16	PIK3CD	23	369.40454	0.225032
17	SIRT1	22	280.5961	0.228792
18	MDM2	22	470.50647	0.230869
19	MTOR	21	230.28436	0.23268
20	PARP1	21	475.82538	0.229086
21	MAPK1	21	534.29694	0.234211
22	IL2	20	579.01013	0.23329
23	PTGS2	20	1893.8696	0.239892
24	ICAM1	20	1011.39825	0.234519
25	CXCR4	19	556.56824	0.225032
26	BCL2L1	19	631.9504	0.236388
27	KIT	18	527.1592	0.23057
28	PPARG	18	678.128	0.234211
29	JAK1	16	39.229153	0.227041
30	LCK	15	209.67973	0.221393
31	ABL1	15	868.95703	0.229381
32	MET	14	209.6729	0.229974
33	CHEK1	14	122.92086	0.211905
34	CDK1	14	326.73175	0.2225
35	IKBKB	14	411.5956	0.220844
36	CHUK	14	400.57013	0.221393
37	PRKCA	13	413.39517	0.223338
38	AR	13	141.84618	0.227331
39	CDC42	13	276.87344	0.227621
40	PDGFRB	12	13.699851	0.218405
41	BRAF	12	117.148224	0.223618
42	SERPINE1	12	434.88385	0.214458
43	TYK2	11	9.84801	0.216282
44	CASP1	11	788.35425	0.222222
45	CHEK2	11	22.461412	0.211653
46	FGFR3	10	76.20105	0.221669
47	PLG	10	253.52045	0.206257
48	TLR9	10	322.4411	0.210651
49	IDH1	10	126.01246	0.221669
50	BRD4	10	23.370459	0.218137
51	CD38	10	177.42128	0.221393
52	FGFR1	10	1001.6348	0.212919
53	PRKDC	10	52.74322	0.22057
54	CYP3A4	10	335.33942	0.193059

55	FLT3	9	32.068314	0.222222
56	CYP1A1	9	192.37564	0.194748
57	PLK1	9	77.19585	0.2109
58	CCR5	9	9.964238	0.209412
59	CCR2	9	11.221954	0.209658
60	CDC25A	9	64.91214	0.213174
61	ALK	9	90.87361	0.220297
62	DNMT1	9	31.373266	0.216809
63	ACE	9	777.5588	0.205069
64	PRKCD	8	39.559155	0.219212
65	F3	8	48.648266	0.199105
66	PKM	8	165.09831	0.221669
67	CYP1A2	8	340.97455	0.208675
68	FLT1	8	100.25979	0.216545
69	CXCR3	8	11.416978	0.203661
70	BTK	8	74.96018	0.204833
71	F2	8	383.24344	0.198218
72	KDM1A	8	11.10389	0.211653
73	APEX1	8	95.53288	0.216282
74	WEE1	8	16.455534	0.213429
75	PPARA	8	439.28036	0.2142
76	HDAC6	8	248.38712	0.21787
77	MAOA	8	1366.9231	0.194111
78	MME	8	232.51787	0.209658
79	SNCA	8	1434.8674	0.217604
80	MMP1	7	37.38858	0.212411
81	PGK1	7	41.410976	0.206977
82	HDAC4	7	17.281921	0.204833
83	GSTP1	7	539.7434	0.209166
84	MAP3K7	7	73.97856	0.206497
85	ITGAL	7	32.57132	0.201586
86	CSF1R	7	10.517397	0.211401
87	NOD2	7	22.345873	0.193268
88	RIPK2	7	17.48334	0.190578
89	NLRP3	7	222.64664	0.204598
90	MPO	7	44.14753	0.200903
91	TERT	7	3.9056072	0.214717
92	G6PD	7	383.22263	0.216019
93	CDK5	7	1108.9879	0.216282
94	YWHAG	7	159.50195	0.214458
95	PLAU	6	15.765765	0.200225
96	FGFR4	6	29.04877	0.210651
97	RET	6	4.25571	0.221945

98	EPAS1	6	36.917843	0.218137
99	EPHX1	6	253.64644	0.185031
100	HPGDS	6	145.19392	0.201586
101	EIF2AK2	6	43.80757	0.217604
102	MDM4	6	10.76658	0.212157
103	EIF2AK3	6	195.39528	0.213685
104	ATR	6	0.2857143	0.20892
105	CYP19A1	6	560.13055	0.211653
106	ANPEP	6	49.326157	0.201586
107	ITGA2B	5	39.27693	0.200225
108	THRB	5	7.1591334	0.215758
109	ITK	5	1.8021834	0.210153
110	IDO1	5	409.28308	0.20892
111	PRF1	5	3.369449	0.197339
112	GRIN2A	5	738.40295	0.184074
113	TYMS	5	143.13712	0.209658
114	NOD1	5	1.7766595	0.187764
115	TOP1	5	1.25	0.20892
116	CTSK	5	140.98036	0.206019
117	CYP2D6	5	48.353302	0.182564
118	SELE	5	38.20253	0.198661
119	XDH	5	677.55347	0.204128
120	RPS27	4	350	0.209166
121	PGD	4	0	0.2
122	HDAC8	4	0	0.214458
123	CSNK2A1	4	25.815598	0.211905
124	COL18A1	4	44.93606	0.197339
125	MMP13	4	27.825514	0.17998
126	P2RX7	4	295.64645	0.187962
127	PRKCE	4	3.5338426	0.205069
128	POLB	4	27.677702	0.208431
129	TGFBR1	4	8.663252	0.196251
130	RAC1	4	0.70316464	0.207218
131	NOS2	4	0.96715784	0.2109
132	ADORA2A	4	729.0187	0.184456
133	ADA	4	258.07535	0.176238
134	ACHE	4	433.8026	0.181078
135	ABCG2	4	452.23474	0.202273
136	ELANE	3	9.455147	0.196685
137	EGLN1	3	1.9233431	0.197998
138	DHFR	3	350	0.208431
139	GRIN2B	3	74.60024	0.187566
140	NEK2	3	0.33333334	0.185224

141	CTSL	3	0	0.20045
142	PARP2	3	0	0.188759
143	DRD2	3	757.201	0.169685
144	DRD1	3	420.08334	0.161818
145	EPHX2	3	49.570904	0.162113
146	ADORA1	3	176.07373	0.162706
147	REN	3	3.3348315	0.186779
148	ATP12A	3	51.96136	0.177291
149	EPHB2	3	7.49659	0.192017
150	CFTR	3	28.272917	0.18896
151	PIK3C2A	2	0	0.193689
152	HLA-DRB1	2	0.5294118	0.194962
153	P2RX3	2	0	0.161965
154	RPS6KA3	2	4.4631066	0.195604
155	FABP4	2	1.794536	0.192432
156	TTR	2	11.5246725	0.184456
157	DUT	2	7.7109666	0.184456
158	SIGMAR1	2	350	0.145663
159	PSEN2	2	11.551038	0.185998
160	CBFB	2	54.54582	0.192225
161	BCHE	2	82.80321	0.175197
162	ALDH2	2	29.220581	0.164207
163	EPHA3	2	0	0.187368
164	RIOK2	1	0	0.173489
165	PTPRF	1	0	0.187171
166	PIM1	1	0	0.200903
167	PDE4D	1	0	0.005618
168	PDE4B	1	0	0.005618
169	GBA	1	0	0.179255
170	SHH	1	0	0.20113
171	DRD5	1	0	0.139608
172	QDPR	1	0	0.172983
173	CYP51A1	1	0	0.127416
174	SHBG	1	0	0.175197
175	CHRNA3	1	0	0.190987
176	ALOX5	1	0	0.194111
177	ACP1	1	0	0.178178
178	CHKA	1	0	0.153713
179	ABCC1	1	0	0.16872

Table S3. The top 10 significant entries of GO Functional enrichment analysis

Category	GO term	Pvalue	Count	GeneRatio	Genes
Biological	Protein	3.97E-12	14	38.89	CHUK, PIK3CD, MTOR, IKBKB, LCK,

process	phosphorylation				ERBB2, CHEK1, CDK1, ABL1, AKT1, MAPK1, JAK2, MET, JAK1
Biological process	Negative regulation of apoptotic process	5.99E-11	13	36.11	SIRT1, MMP9, EGFR, IL2, MTOR, IKBKB, ERBB2, MDM2, BCL2, CDK1, AKT1, TP53, BCL2L1
Biological process	Positive regulation of transcription from RNA polymerase II promoter	8.71E-10	16	44.44	PARP1, CHUK, STAT1, STAT3, HIF1A, SIRT1, EGFR, IL2, IKBKB, ABL1, AKT1, EP300, PPARG, JAK2, MET, TP53
Biological process	Peptidyl-tyrosine phosphorylation	2.64E-09	7	19.44	PTPRC, LCK, ERBB2, KIT, ABL1, JAK2, EGFR
Biological process	Cellular response to hypoxia	3.65E-09	8	22.22	MDM2, BCL2, PPARG, PTGS2, HIF1A, SIRT1, TP53, MTOR
Biological process	Apoptotic process	6.27E-09	12	33.33	PARP1, CHEK1, MDM2, BCL2, CDK1, EP300, MAPK1, CXCR4, JAK2, TP53, MMP9, BCL2L1
Biological process	Protein autophosphorylation	2.72E-08	8	22.22	PTPRC, ERBB2, KIT, ABL1, AKT1, JAK2, EGFR, MTOR
Biological process	Negative regulation of autophagy	6.60E-08	6	16.67	STAT3, BCL2, AKT1, MET, MTOR, BCL2L1
Biological process	T cell receptor signaling pathway	1.52E-07	7	19.44	IKBKB, PTPRC, PIK3CA, LCK, ABL1, MAPK1, PIK3CD
Biological process	Peptidyl-threonine phosphorylation	1.57E-07	6	16.67	CHEK1, BCL2, CDK1, MAPK1, AKT1, MTOR
Cellular component	Macromolecular complex	4.03E-11	14	38.89	PARP1, STAT1, CXCR4, PTPN11, PTGS2, HIF1A, SIRT1, EGFR, CHEK1, MDM2, BCL2, ABL1, AKT1, TP53
Cellular component	Cytosol	1.01E-09	28	77.78	CXCR4, PIK3CD, HIF1A, IKBKB, ERBB2, CHEK1, ABL1, AKT1, MAPK1, EP300, JAK2, JAK1, PARP1, CHUK, STAT1, STAT3, PTPN11, SIRT1, MTOR, PIK3CA, LCK, BCL2, CDK1, MDM2, PPARG, GAPDH, TP53, BCL2L1
Cellular component	Cytoplasm	1.51E-09	28	77.78	CXCR4, PIK3CD, PTGS2, HIF1A, EGFR, IKBKB, CHEK1, ABL1, AKT1, MAPK1, EP300, JAK2, JAK1, PARP1, CHUK, STAT1, STAT3, PTPN11, SIRT1, MTOR, PIK3CA, BCL2, CDK1, MDM2, PPARG, GAPDH, TP53, BCL2L1
Cellular component	Nucleus	1.70E-06	25	69.44	CXCR4, HIF1A, EGFR, IKBKB, ERBB2, CHEK1, ABL1, AKT1, MAPK1, EP300, JAK2, JAK1, PARP1, CHUK, STAT1, STAT3, PTPN11, SIRT1, MTOR, BCL2, CDK1, MDM2, PPARG, GAPDH, TP53
Cellular	Nucleoplasm	5.12E-06	20	55.56	PARP1, CHUK, STAT1, STAT3, PTPN11,

component					HIF1A, SIRT1, MTOR, ERBB2, CHEK1, MDM2, BCL2, CDK1, ABL1, AKT1, EP300, MAPK1, PPARG, JAK2, TP53
Cellular component	Cytoplasmic side of plasma membrane	9.54E-06	5	13.89	IKBKB, PTPRC, CHUK, KIT, JAK1
Cellular component	Membrane raft	5.51E-05	6	16.67	IKBKB, PTPRC, LCK, JAK2, EGFR, ICAM1
Cellular component	Extrinsic component of cytoplasmic side of plasma membrane	2.57E-04	4	11.11	LCK, AKT1, JAK2, JAK1
Cellular component	Chromatin	3.08E-04	9	25.00	PARP1, STAT1, STAT3, CHEK1, EP300, PPARG, HIF1A, SIRT1, TP53
Cellular component	Receptor complex	5.09E-04	5	13.89	ERBB2, KIT, PPARG, MET, EGFR
Molecular function	Protein serine/threonine/tyrosine kinase activity	1.61E-14	15	41.67	EGFR, MTOR, IKBKB, PIK3CA, LCK, KIT, ERBB2, CHEK1, CDK1, ABL1, AKT1, MAPK1, JAK2, MET, JAK1
Molecular function	Identical protein binding	1.21E-11	20	55.56	PARP1, STAT1, STAT3, SIRT1, MMP9, EGFR, MTOR, IKBKB, LCK, ERBB2, MDM2, BCL2, AKT1, MAPK1, PPARG, JAK2, GAPDH, MET, TP53, BCL2L1
Molecular function	Protein phosphatase binding	2.60E-10	8	22.22	LCK, ERBB2, STAT3, PPARG, TP53, MET, EGFR, JAK1
Molecular function	Protein kinase activity	1.05E-09	11	30.56	IKBKB, CHUK, ERBB2, CHEK1, CDK1, ABL1, AKT1, JAK2, MET, EGFR, MTOR
Molecular function	Protein tyrosine kinase activity	1.30E-09	8	22.22	LCK, ERBB2, KIT, ABL1, JAK2, MET, EGFR, JAK1
Molecular function	Protein kinase binding	1.43E-09	12	33.33	IKBKB, PTPRC, PARP1, LCK, STAT3, ABL1, AKT1, PTPN11, JAK2, HIF1A, TP53, BCL2L1
Molecular function	ATP binding	3.17E-09	17	47.22	CHUK, PIK3CD, EGFR, MTOR, IKBKB, PIK3CA, LCK, KIT, ERBB2, CHEK1, CDK1, ABL1, AKT1, MAPK1, JAK2, MET, JAK1
Molecular function	Kinase activity	7.17E-09	9	25.00	PIK3CA, ERBB2, CDK1, ABL1, PIK3CD, AKT1, JAK2, EGFR, MTOR
Molecular function	Enzyme binding	2.13E-08	10	27.78	PARP1, STAT1, MDM2, AKT1, PPARG, PTGS2, HIF1A, SIRT1, TP53, EGFR
Molecular function	Protein binding	7.11E-07	36	100.00	CXCR4, PIK3CD, PTGS2, HIF1A, EGFR, ICAM1, IKBKB, ERBB2, CHEK1, ABL1, AKT1, MAPK1, EP300, JAK2, JAK1, PARP1, CHUK, STAT1, STAT3, PTPN11, MMP9, SIRT1, IL2, MTOR, PTPRC, PIK3CA, LCK, KIT, BCL2, CDK1,

					MDM2, PPARG, MET, GAPDH, TP53, BCL2L1

Table S4. The top 15 significant entries of KEGG pathway enrichment analysis

Pathway	PValue	Count	GeneRatio	Genes
PI3K-Akt signaling pathway	5.71E-15	18	50.00	CHUK, PIK3CD, EGFR, IL2, MTOR, IKBKB, PIK3CA, KIT, ERBB2, MDM2, BCL2, AKT1, MAPK1, JAK2, MET, TP53, JAK1, BCL2L1
JAK-STAT signaling pathway	4.01E-14	14	38.89	STAT1, STAT3, PIK3CD, PTPN11, EGFR, IL2, MTOR, PIK3CA, BCL2, AKT1, EP300, JAK2, JAK1, BCL2L1
Chronic myeloid leukemia	2.83E-13	11	30.56	IKBKB, PIK3CA, CHUK, MDM2, ABL1, MAPK1, PIK3CD, AKT1, PTPN11, TP53, BCL2L1
HIF-1 signaling pathway	3.11E-13	12	33.33	PIK3CA, ERBB2, STAT3, BCL2, EP300, MAPK1, PIK3CD, AKT1, HIF1A, GAPDH, EGFR, MTOR
C-type lectin receptor signaling pathway	7.18E-12	11	30.56	IKBKB, PIK3CA, CHUK, STAT1, MDM2, MAPK1, PIK3CD, AKT1, PTPN11, PTGS2, IL2
FoxO signaling pathway	7.37E-11	11	30.56	IKBKB, PIK3CA, CHUK, STAT3, MDM2, EP300, MAPK1, PIK3CD, AKT1, SIRT1, EGFR
Acute myeloid leukemia	1.99E-10	9	25.00	IKBKB, PIK3CA, CHUK, STAT3, KIT, MAPK1, PIK3CD, AKT1, MTOR
Thyroid hormone signaling pathway	9.54E-10	10	27.78	PIK3CA, STAT1, MDM2, EP300, MAPK1, PIK3CD, AKT1, HIF1A, TP53, MTOR
Ras signaling pathway	1.45E-09	12	33.33	IKBKB, PIK3CA, CHUK, KIT, ABL1, MAPK1, PIK3CD, AKT1, PTPN11, MET, EGFR, BCL2L1
Apoptosis	2.71E-09	10	27.78	IKBKB, PIK3CA, CHUK, PARP1, BCL2, MAPK1, PIK3CD, AKT1, TP53, BCL2L1
AGE-RAGE signaling pathway in diabetic complications	5.17E-09	9	25.00	PIK3CA, STAT1, STAT3, BCL2, MAPK1, PIK3CD, AKT1, JAK2, ICAM1
T cell receptor signaling pathway	7.07E-09	9	25.00	IKBKB, PTPRC, PIK3CA, CHUK, LCK, MAPK1, PIK3CD, AKT1, IL2
TNF signaling pathway	1.47E-08	9	25.00	IKBKB, PIK3CA, CHUK, MAPK1, PIK3CD, AKT1, PTGS2, MMP9, ICAM1
Neurotrophin signaling pathway	2.06E-08	9	25.00	IKBKB, PIK3CA, BCL2, ABL1, MAPK1, PIK3CD, AKT1, PTPN11, TP53
ErbB signaling pathway	4.29E-08	8	22.22	PIK3CA, ERBB2, ABL1, MAPK1, PIK3CD, AKT1, EGFR, MTOR

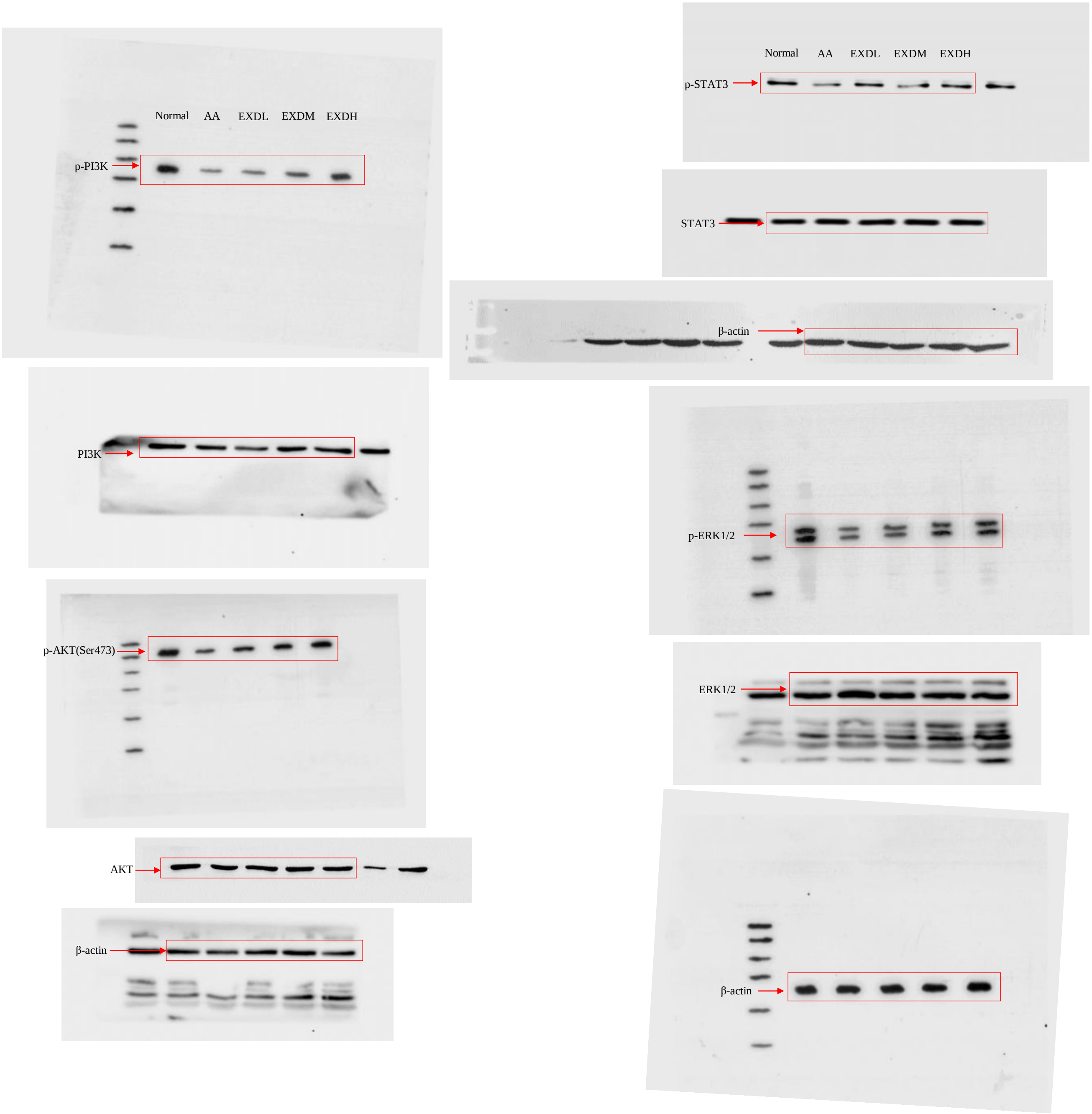

Figure S1. The full-length images of western blot
